# Proinflammatory cytokines correlate with early exercise attenuating anxiety‐like behavior after cerebral ischemia

**DOI:** 10.1002/brb3.854

**Published:** 2017-10-18

**Authors:** Qi Zhang, Jingjun Zhang, Yuzhong Yan, Pengyue Zhang, Wei Zhang, Rong Xia

**Affiliations:** ^1^ Department of Blood Transfusion Huashan Hospital Fudan University Shanghai China; ^2^ Medical Faculty Kunming University of Science and Technology Kunming China; ^3^ Department of Medical Imaging Renji Hospital Medical School of Jiaotong University Shanghai China

**Keywords:** anxiety‐like behavior, early exercise, immune activation, ischemia, proinflammatory cytokines

## Abstract

**Background and Objective:**

Stroke may cause neuropsychiatric problems, which have negative effects on cognitive functions and behavior. Exercise plays an important role in reducing the occurrence and development of stroke, the concrete mechanism is not fully clarified. In this study, we attempted to determine whether early treadmill exercise attenuates anxiety‐like behavior by regulation of inflammation after brain ischemia.

**Method:**

We subjected adult male rats to middle cerebral artery occlusion (MCAO) for 90 min and trained rats started to run on a treadmill from postoperative day 1 to day 14. The effects of treadmill on cognitive functions, anxiety‐like behavior, and immune activation were analyzed by Morris water maze test, open field test, elevated plus maze test, and enzyme‐linked immunosorbent assay.

**Results:**

Early treadmill exercise significantly improved cognitive function, alleviated anxiety‐like behavior in ischemic rats model; this improvement was associated with significantly decreased activation of astrocytes and microglia cells and proinflammatory markers (platelet‐activating factor [PAF], interleukin‐6 [IL‐6], tumor necrosis factor‐alpha [TNF‐α], intercellular adhesion molecule‐1 [ICAM‐1], and vascular cell adhesion molecule‐1 [VCAM‐1]).

**Conclusion:**

Our results indicated that early treadmill exercise attenuated anxiety‐like behavior by decreasing inflammation response, exercise conferred a great benefit of attenuating anxiety‐like behavior via anti‐inflammatory treatment may prove to be a novel neuroprotective strategy for stroke.

## 1 INTRODUCTION

Stroke is a major refractory disease significantly threatening human health and life with high morbidity, disability, and mortality (Lang & Borgwardt, 2013). The incidence of stroke increases annually by 9% and has been a leading cause of death in China. Mood disturbance is common complication of stroke and is characterized by mood abnormalities, self‐blaming, sadness, especially anxiety and depression (Lang & Borgwardt, 2013). Poststroke depression (PSD) has received most research attention (Wu et al., 2014), in contrast, poststroke anxiety (PSA) has only recently gained attention although anxiety is commoner than depression (Menlove et al., 2015).

Recent studies reveal that exercise plays an important role in reducing the occurrence and development of chronic metabolic and cardiorespiratory diseases, as well as stroke. Regular exercise reduces the risk of stroke, in part because exercise exerts anti‐inflammatory effects on rats (Batista et al., 2010; Eng & Reime, 2014). On the other hand, mood disorder is associated with a chronic, low‐grade inflammatory response (Eng & Reime, 2014; Yan, Fang, & Liu, 2013), change in expressions of proinflammatory mediators in rats plasma is associated with disease progression (Lu et al., 2014; Noto et al., 2014). In mood disorders patients’ sera such as anxiety, the levels of proinflammatory cytokines were increased (Menlove et al., 2015; Pláteník et al., 2014), sustained production of inflammatory cytokines in transgenic mice is accompanied by the onset of depression‐like behavior (Noto et al., 2014).

After considering the role of exercise on the immune system response and the effect of inflammation on the mood disorder progression, the present study focused on neuroinflammatory mechanism and mood disturbance involved in the early exercise strategy against stroke, we examined the effects of early exercise on anxiety‐like behavior, cognitive function, and levels of proinflammatory responses in a middle cerebral artery occlusion (MCAO) rat model. Our study may extend the present knowledge of the mechanisms underlying exercise induced neuroprotective effect in the stroke rats.

## 2 METHODS

### 2.1 Animals and treadmill training

Male Sprague‐Dawley rats (weighing 250–280 g) provided by the Shanghai Laboratory Animal Center, Chinese Academy of Sciences and were housed under a under a 12:12 h light:dark cycle at 21 ± 1°C with ad libitum access to food and water. Animals were divided into three experimental groups: (i) sham (S); (ii) ischemia (I); and (iii) ischemia‐exercise (IE) (*n* = 15 for each group: *n* = 5 for two anxiety‐like behavior tests and ELISA analysis at day 7 and another five rats for these tests at day 14; *n* = 5 for Morris water maze [MWM] test at day 14). Animals in the ischemia‐exercise groups exercised on a rat treadmill (DSPT‐202 Type 5‐Lane treadmill; Litai Biotechnology Co., Ltd., China) from postoperation days 1–14, for a total of 14 days at 30 min/day. On the first and second exercise days, the treadmill velocity was 5 m/min for the first 10 min, 9 m/min for the second 10 min, 12 m/min for the last 10 min, and 12 m/min on the third and subsequent days. The tilt angle was 0°. Animals in the sham and ischemia group were housed freely in cages. To reduce the stress of treadmill training, all rats were adapted to the treadmill at a speed of 6 m/min for three consecutive days (10 min/day) before the operation. All procedures were performed according to the guidelines of the National Institutes of Health Guide for the Care and Use of Laboratory Animals.

### 2.2 Middle cerebral artery occlusion model

Rats were anesthetized with 1.5% isoflurane (Abbott, Abbott Park, IL, USA) and the left middle cerebral artery was occluded by the intraluminal suture technique as previously described (Longa, Weinstein, Carlson, & Cummins, 1989; Zhang, Yu, et al., 2013). Briefly, the middle cerebral artery was occluded by a 4‐0 nylon monofilament coated with a silicone tip. Reperfusion was established by gently withdrawing the filament after 90 min of occlusion. In the sham group, all of the surgical procedures were included except for the occlusion of the MCA.

### 2.3 Morris water maze test

To assess the spatial learning and memory ability MWM began at day 14 after operation (Zhang et al., 2012). Briefly, the water maze consisted of a black circular tank containing with water and situated in a room with salient visual cues. Acquisition of spatial learning was performed for four consecutive days. A platform was submerged 2 cm underneath the water in the middle of one‐quadrant of the tank. Each rat received five trials (with randomly assigned starting positions) per day to locate the platform with an inter‐trial interval of 10 s. The rat was given a maximum time of 60 s to locate the platform after which the rat remained there for 10 s. If it did not locate the platform within 60 s, the rat was manually guided to the platform. The mean escape latency per day was recorded for each animal. A spatial probe test was performed on the fifth day to evaluate memory retention of the rats. The rats were placed into the tank at the most distal location to the target quadrant where the platform had been previously located. The percentage of time spent in the target quadrant was recorded (Cheng et al., 2013).

### 2.4 Open field test

The free exploration of a square open field (60 cm for length, width, and height) was analyzed for 5 min after 7 and 14 days of treadmill exercise. The apparatus was further divided into two parts, the center (35 cm for length and width) and the periphery. The examined behaviors included the distance and the time of exploration of the center and the periphery (Aguiar et al., 2014).

### 2.5 Elevated plus maze

The equipment consisted of two opposite arms (50 × 15 cm) which were connected with a central square (12 × 12 cm), the apparatus shape was a plus sign. One arm remained open, while the other arm was enclosed with a 40 cm elevated wall. The whole apparatus was elevated 120 cm from the ground. Rats were located individually in the center of the cross, facing an enclosed arm, and the time spent in the open arms were recorded during a 5‐min test period after 7 and 14 days of treadmill exercise. More time spent in open arm indicated non‐anxiety behavior.

### 2.6 Blood sample collection

At the completion of behavioral testing at days 7 and 14, all rats were deeply anesthetized, and the blood samples (2 mL) were collected from the jugular vein using a tube containing ethylene diaminetetraacetic acid (EDTA) and aprotinin. All blood samples were centrifuged at 1,250 *g* for 10 min at 4°C, and the supernatants were stored at −80°C until use.

### 2.7 Immunofluorescence staining

The primary antibodies used in this study were anti‐glial fibrillary acidic protein (GFAP, Cell Signalling Technology, USA) and anti‐ionized calcium‐binding adapter molecule 1 (Iba‐1, Wako, Japan). Brain sections (30 μm thickness) on days 7 and day 14 post‐ischemic/reperfusion injury were treated with 1% Triton X‐100 for 20 min before blocking in 10% goat serum for 1 h. Sections were then incubated with primary antibodies for 1 h at 37°C and then overnight at 4°C. After washing, slides were further incubated at 37°C for 2 h, with the following secondary antibodies: either DyLightTM 594‐conjugated goat anti‐mouse or DyLightTM 488‐conjugated goat anti‐rabbit (Jackson ImmunoResearch Laboratories, USA), followed by counterstained with DAPI (Thermo Scientific, USA) for 2 min at room temperature. For densitometric analysis‐based NIH Image‐analysis system software was used as previously described (Liu, Silverstein, Skoff, & Barks, 2002). Briefly, three sections (bregma 1.0 mm) were obtained from each animal and three randomly selected areas in ischemic penumbra were digitized to images using the same exposure time. The images were then binarized and segmented under a consistent threshold (50%). Next, the total black pixels per image were counted. To minimize the differences in the fluorescent intensity among the immunostained sections, pixels values were calculated as ratios of injury in the ipsilateral (IL) relative to the contralateral (CL) hemisphere (IL: CL = lesion: intact hemisphere).

### 2.8 Determination of inflammatory mediators levels in plasma

The levels of platelet‐activating factor (PAF), interleukin‐6 (IL‐6), tumor necrosis factor‐alpha (TNF‐α), intercellular adhesion molecule‐1 (ICAM‐1), and vascular cell adhesion molecule‐1 (VCAM‐1) in plasma were assayed using an enzyme‐linked immunosorbent assay kit (ELISA [Kit Xitang from Shanghai Biotechnology Co., Ltd.]) following the manufacturer's instructions.

### 2.9 Statistical analysis

All data were presented as the means ± standard error of the mean (SEM). Parametric analysis of variance (ANOVA) followed by LSD posthoc test was applied for mean comparisons. Correlations between plasma cytokine levels and the anxiety‐like behavior were calculated and analyzed using Spearman's correlation coefficient. *p*‐values <.05 were considered statistically significant.

## 3 RESULTS

### 3.1 Early exercise improved spatial learning and memory

**Figure 1:**
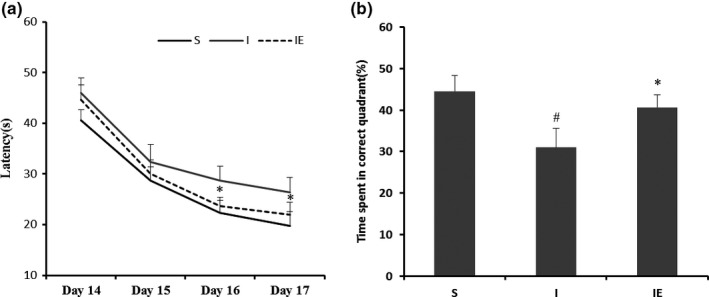
Exercise‐treatment improves cognitive functions in MCAO rats. (a) Evaluation of spatial learning using the Morris water maze latency test at 14 days after cerebral ischemia for four consecutive days (Day 14–Day 17). There was no significant difference between the S and IE groups in terms of spatial learning (*p < .05 vs. I group). (b) Determination of spatial memory using the probe test 1 day after the final acquisition training session. The submerged platform used in the previous test was removed, and the percent of time spent in the quadrant where the platform was previously located was measured. Ischemia group rats showed significant impairment in this task, whereas Ischemia‐exercise group rats performed at the level of sham rats (*n* = 10 rats per group, ^#^ *p* < .05 vs. S group, *p < .05 vs. I group)

Morris water maze were designed to study learning and memory performance in MCAO rats with treadmill treatment. The initial assessment of spatial learning revealed that ischemia rats showed a significant increase in latency to find the platform on days 3 and 4 of training (days 16 and 17 after operation, respectively) compared to both the sham and exercise group (Figure 1a). There was no statistical difference between sham and exercise group in spatial learning. In the spatial probe test trials, results indicated that IE group rats spent more time (40.69 ± 2.96%) in the target quadrant than the I group rats (30.98 ± 4.69%, *n* = 10, *p* < .01, Figure 1b), suggesting that treadmill treatment was effective in attenuating spatial memory deficits in MCAO rats. Collectively, results from the MWM experiments indicated that cognitive impairments in MCAO rats are ameliorated by exercise.

### 3.2 Early exercise ameliorated anxiety‐like behavior

**Figure 2:**
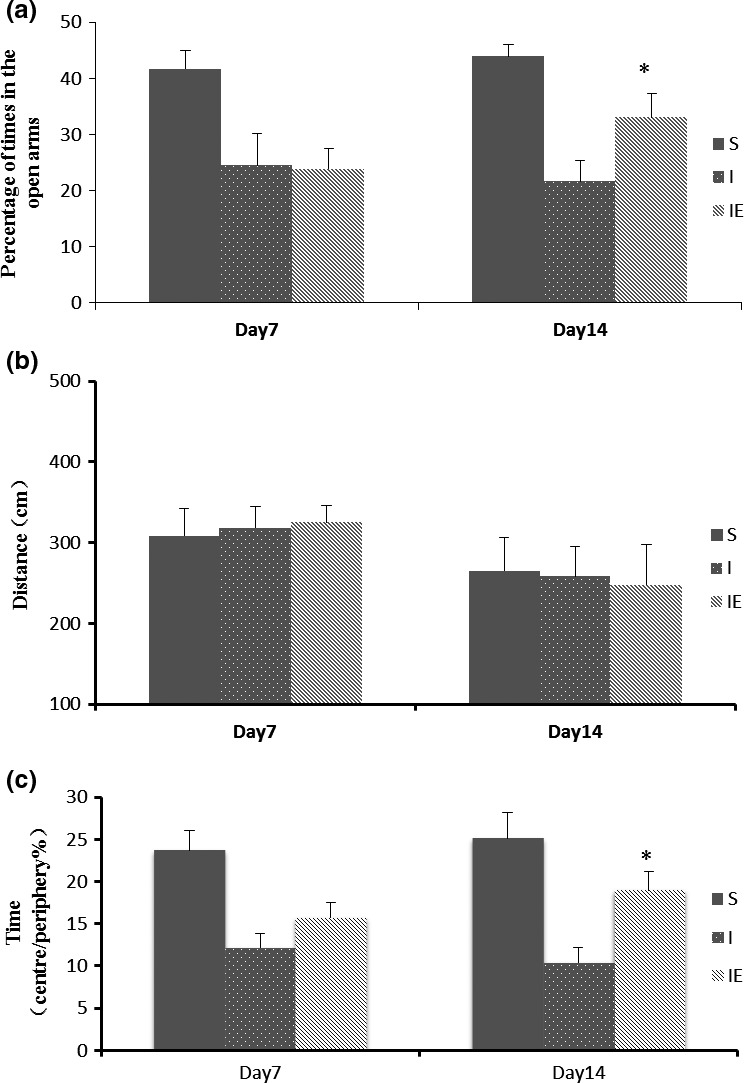
Exercise attenuates poststroke anxiety‐like behavior. (a) Results of elevated plus maze test. The rats spent time in the open arm was observed. Compared to the ischemia (I) group, the rats in the exercise group (IE) had spent more time in open arm at day 14. (b,c) Results of open field test. Compared to the ischemia (I) group, the rats in the exercise group (IE) had spent more exploration time of central regions at day 14. *p < .05, versus I group, *n* = 10 rats per group

The results of the elevated plus maze (Figure 2a) showed that the time spent in the open arm differed significantly between ischemia and exercise group at day 14 (*F* = 11.65, *p* < .01), and no difference was observed among groups at day 7. More time spent in open arm indicated non‐anxiety behavior, so the results indicated anxiety‐like behavioral were improved in the exercise group.

All spontaneous locomotor activities of the animals were observed (Figure 2b). Results showed that the groups were not significantly different at day 7 and day 14, but statistically significant differences of exploration time of central regions were observed between the treadmill treatment and ischemia model group at day 14 (Figure 4c). The results indicated that treadmill treatment rats showed an increased exploration of central regions of the open field.

### 3.3 Early exercise inhibited the activation of glial cells

**Figure 3:**
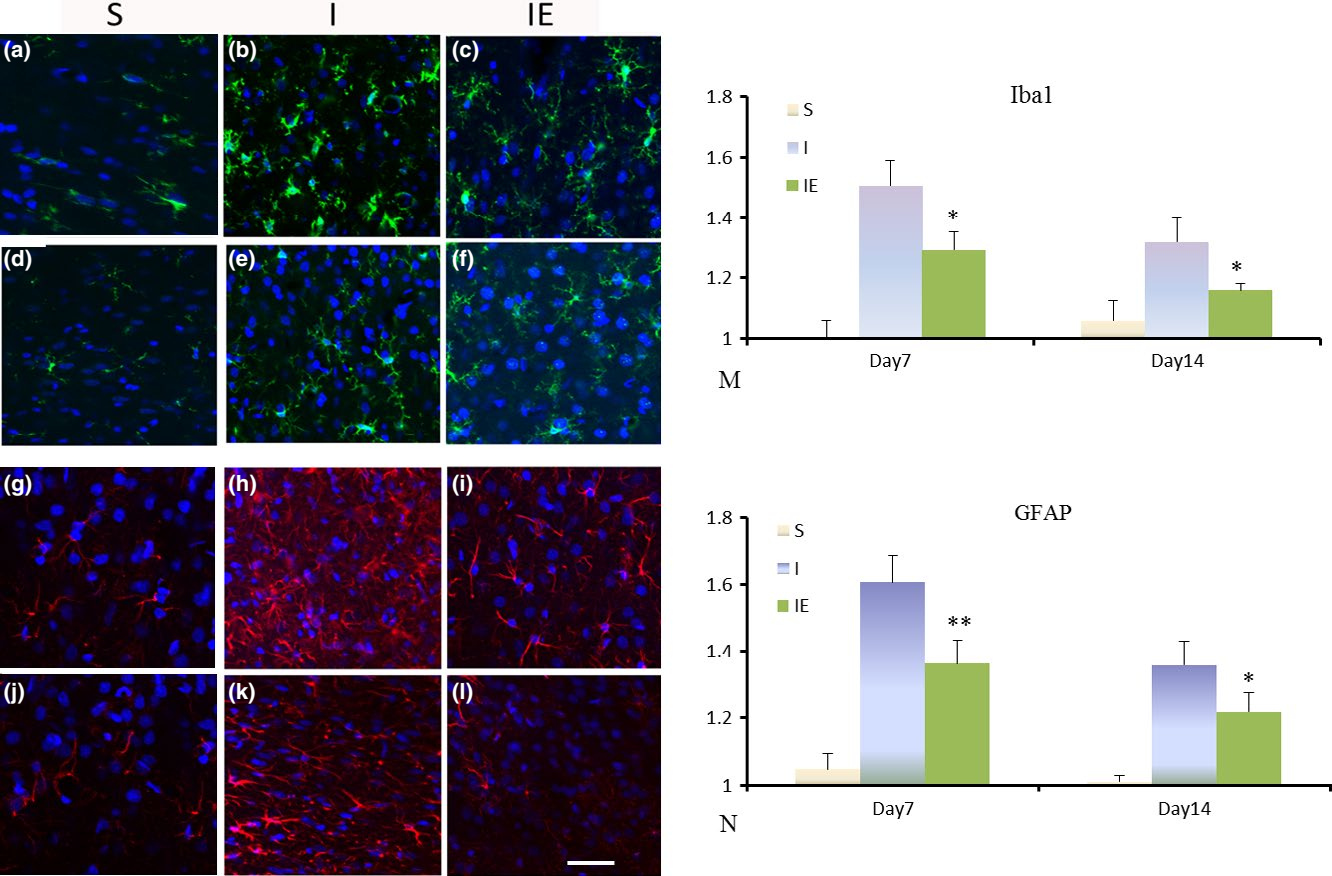
Exercise inhibited the activation of astrocytes and microglia cells. Representative immunofluorescence photomicrographs of Iba1 (green, a–f) and GFAP (red, g–l) in the prefrontal cortex at day 7 (a–c, g–i) and 14 (d–f, j–l) post‐cerebral ischemia. Dapi stain of nuclei is depicted in blue. Quantification of the densitometric analysis of Iba1 (m) and GFAP (n) immunostaining demonstrates that exercise significantly decreased reactive microglial and astrocytosis in prefrontal cortex examined at both days 7 and 14 post‐ischemia. *p < .05, **p < .01 versus the NE group. *n* = 5 rats per group, Scale bar = 50 μm

To investigate whether treadmill treatment had an effect on glial activation, immunofluorescence analysis was used to measure the activation of GFAP and Iba1. Brain section staining showed that reactive astrocytes were observed at the peri‐lesional regions in prefrontal cortex. Exercise‐treatment significantly decreased ramified GFAP‐immunoreactive astrocytic at days 7 and 14 post‐ischemia. Activated microglias were also detected in the prefrontal cortex. Treadmill exercise significantly reduced the ratio of activated microglia compared with the IE group rats in the cortex at days 7 and 14 post‐ischemia. These data indicated that glial activation induced by ischemia could be decreased by exercise‐treatment (Figure 3).

### 3.4 Early exercise‐induced changes in inflammatory substances

**Figure 4:**
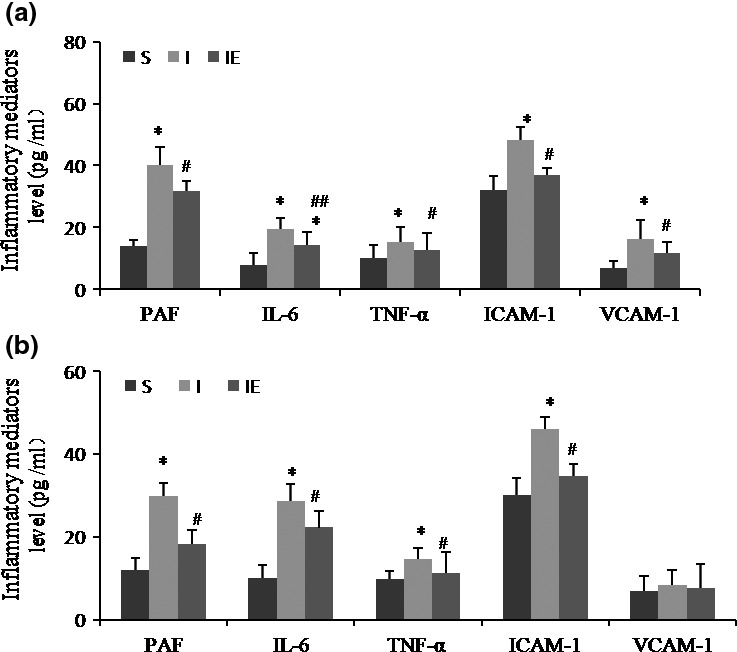
Exercise attenuated inflammatory substances expression after ischemia. Inflammatory mediators (PAF, IL‐6 TNF‐α, ICAM, and VCAM) expression at day 7 (a) and day 14 (b) after ischemia. Note that expression levels of most inflammatory cytokines were attenuated by treadmill exercise. *p < .05, versus S group. ^#^ *p* < .05, ^##^ *p* < .01 versus I group, *n* = 5 rats per group

To evaluate the effect of treadmill treatment on ischemia‐induced inflammation, we studied the expression of inflammatory mediators at day 7 and 14 post‐ischemia. At all test points, the PAF and proinflammatory cytokines IL‐6, TNF‐α levels were significantly decreased by exercise‐treatment. On day 7, treadmill treatment markedly decreased the aforementioned cytokines expression compared to the ischemia rats (Figure 4a). At day 14 post‐ischemia, the levels of IL‐6 and TNF‐α in the exercise‐treatment group remained significantly lower than ischemia group (Figure 4b).

ICAM‐1 and VCAM‐1 are the adhesive molecules that also play a regulatory role during inflammation, mediated leukocyte migration into the injured CNS, and contribute to ischemia‐induced neuroinflammation. We found the expression of ICAM‐1 and VCAM‐1 was significantly decreased in the IE group compared with I group at 7 and 14 days. The results demonstrated that inflammatory mediators were decreased in MCAO rats with treadmill treatment.

### 3.5 Correlations of anxiety‐like behavior with plasma cytokine levels

Correlational analyses revealed a number of significant correlations between cytokines and physical symptom of anxiety. A strongly negative correlation was observed between cytokines (PAF, IL‐6, and TNF‐α) and anxiety‐like behavior (time spent in open arm and exploration time of central regions). No correlation was found between plasma concentrations of other cytokines (ICAM‐1 and ICAM‐1) and anxiety‐like behavior (Table 1).

**Table 1:** Correlation between levels of cytokines and anxiety‐like behavior

	PAF	IL‐6	TNF‐α	ICAM‐1	VCAM‐1
Time spent in open arm	−.44**	−.22	−.48**	−.19	−.15
Exploration time of central regions	−.34*	−.40*	−.31*	−.11	−.10

Values represent the correlation coefficients (*R*‐values).

Correlation is significant at *p <* *.05, *^*^ *p *<* *.01.

## 4 DISCUSSION

Recent ample evidence suggests that physical rehabilitation within 24 h after stroke may improve neurologic recovery in preclinical and clinical studies, and the underlying mechanism for very early initiated exercise‐afforded neuroprotection has not yet been fully elucidated. In the present study, we confirmed that early treadmill exercise improved anxiety‐like behavior and learning and memory and induced decreases in glial cells and proinflammatory cytokines (PAF, IL‐6, TNF‐α, ICAM, and VCAM) activation. What's more, we found significant correlations between cytokines and physical symptom of anxiety. In summary, a possible mechanism for early exercise‐induced neuroprotection against PSA was through regulation of inflammation to promote neurologic recovery and ameliorate anxiety‐like behavior, and proinflammatory cytokines (PAF, IL‐6, and TNF‐α) may play a critical role in the process.

Anxiety disorders or symptoms is a common complication after stroke and is accompanied with cognitive decline or dementias (Lang & Borgwardt, 2013). The prevalence of PSA ranges between 20% within 1 month after stroke and 24% 6 months or more after stroke (Menlove et al., 2015). Most importantly, PSA not only increases the difficulties associated with the patient's care (by both family and society), but also reduces the patient's compliance with stroke rehabilitation (Lang & Borgwardt, 2013; Zhang, Yu, et al., 2013). Clinical and neurobiological data suggest that exercise may be an effective treatment strategy to ease the symptoms of anxiety disorders (Hennings et al., 2013). Previous studies demonstrated that aerobic exercise could reduce depression and state anxiety in clinical populations, exercise may also reduce the likelihood of new depressive and anxiety disorders in older individuals (Weinstein, Maayan, & Weinstein, 2015). Our studies focused on the effects of early exercise on anxiety‐like behavior after stroke and showed that exercise‐treatment attenuated PSA‐like behavior as measured by open field test and elevated plus maze test. Our results provided a new context for protecting stroke patients from anxiety.

Inflammation contributes to the pathophysiology of anxiety and stroke in a subset of the clinical population. Following stroke, the acute inflammation appears within hours and persists for days. Excess levels of inflammatory mediators occur in a subgroup of depressed and anxious patients (Kamo, Ke, Busuttil, & Kupiec‐Weglinski, 2013; Maldonado‐Bouchard et al., 2016), and the inappropriate glial activity could cause anxiety through deleterious effects on neuroplasticity (Zhu et al., 2016). Our study found early exercise suppressed glial activity and proinflammatory cytokine expression as well as reducing anxiety‐like behavior. Anxiety‐associated proinflammatory cytokines released from immune cells, as a result of inflammation or stress, have abnormal serum levels in these patients (Pandey, Rizavi, Ren, Bhaumik, & Dwivedi, 2014; Toscano et al., 2016), and increased levels of cytokines contribute to the progress of depression and anxiety (Maldonado‐Bouchard et al., 2016). Our data showed exercise not only reduced the expression of inflammatory cytokines (IL‐6, TNF‐α) of MCAO rats significantly, but also reduced the expression of cell adhesion molecules (ICAM and VCAM), which mediated the leukocyte adhesion and infiltration in response to ischemia reperfusion injury. TNF‐α was elevated in the hippocampi of the anxiety groups, IL‐6 and TNF‐α were also significantly increased in the plasma of the subjects with signs of anxiety. In our correlational analyses, a negative association was observed between IL‐6 and TNF‐α levels and time spent in open arm and exploration time of central regions and indicated that proinflammatory cytokines may be involved in the pathophysiology of anxiety‐like behavior.

Platelet‐activating factor is a powerful short‐lived chemical mediator of leukocyte functions, platelet aggregation, and neutrophil activation (Mazereeuw et al., 2013). During ischemia, PAF increases and then becomes a proinflammatory messenger via modulating leukocyte‐endothelium adhesion. Excessive PAF promotes neuronal damage by disruption of the blood–brain barrier, reduction of cerebral blood flow, and stimulation of leucocytes. In the presence of brain injury, inflammatory activation may serve as the linchpin that precipitates dysregulation of biologic systems leading to changes to behavior (Toscano et al., 2016). PAF is the potent inflammatory mediator involved in various inflammatory diseases including mood disorder. In the current study, we not only found exercise decreased PAF expression in plasma, but also found a negative correlation between PAF and anxiety‐like behavior. Numerous studies suggest that PAF induces IL‐6 and TNF‐α release from leukocytes and platelets, PAF induces surface expression of ICAM‐1 and VCAM‐1, facilitating leukocyte adhesion to endothelial cells (Mazereeuw et al., 2013; Yoshida et al., 2005). Previous studies also showed that PAF receptor antagonists repress the chemotaxis of inflammatory cells, and to inhibit the inflammatory response in neural cells (Maldonado‐Bouchard et al., 2016). Collectively, prior works as well as our current data indicated that PAF may be the regulator of decreased activation of glial cells and expression of proinflammatory cytokines and contributed to exercise‐induced alleviating anxiety‐like behavior.

In conclusion, our results provided novel evidence that early exercise‐associated decrease in neuroinflammation accompanied by a reduction in anxiety‐like behavior and cognitive impairment after the stroke. Based on these findings, we proposed that early exercise attenuated inflammation directly contributing to improved neuroprogression in PSA, and proinflammatory cytokines (PAF, IL‐6, and TNF‐α) may be the key factors involved in the neuroinflammatory mechanism of exercise strategy against PSA. Hopefully, this will encourage others to further investigate the neuroprotection of early exercise for PSA and other stroke‐related disease.

## CONFLICT OF INTERESTS

None declared.
